# Low vitamin D and the risk of developing chronic widespread pain: results from the European male ageing study

**DOI:** 10.1186/s12891-016-0881-6

**Published:** 2016-01-16

**Authors:** Paul S. McCabe, Stephen R. Pye, John Mc Beth, David M. Lee, Abdelouahid Tajar, Gyorgy Bartfai, Steven Boonen, Roger Bouillon, Felipe Casanueva, Joseph D. Finn, Gianni Forti, Aleksander Giwercman, Ilpo T. Huhtaniemi, Krzysztof Kula, Neil Pendleton, Margus Punab, Dirk Vanderschueren, Frederick C. Wu, Terence W. O’Neill, for the EMAS Study Group

**Affiliations:** Arthritis Research UK Centre for Epidemiology, Centre for Musculoskeletal Research, Institute of Inflammation and Repair, University of Manchester, Stopford Building, Oxford Road, M13 9PT Manchester, UK; Arthritis Research Primary Care Centre, Primary Care Sciences, Keele University, Keele, UK; Department of Obstetrics, Gynaecology and Andrology, Albert Szent-Gyorgy Medical University, Szeged, Hungary; University Division of Geriatric Medicine, Katholieke Universiteit Leuven, Leuven, Belgium; Department of Experimental Medicine, Katholieke Universiteit Leuven, Leuven, Belgium; Department of Medicine, Santiago de Compostela University, Complejo Hospitalario Universitario de Santiago, CIBER de Fisiopatología Obesidad y Nutricion, Instituto Salud Carlos III, ; Santiago de Compostela, Spain; Andrology Research Unit, Centre for Endocrinology and Diabetes, University of Manchester, Manchester, UK; Andrology Unit, Department of Clinical Physiopathology, University of Florence, Florence, Italy; Scanian Andrology Centre, Department of Urology, Malmö University Hospital, University of Lund, Lund, Sweden; Department of Reproductive Biology, Imperial College London, London, UK; Department of Andrology and Reproductive Endocrinology, Medical University of Lodz, Lodz, Poland; Clinical Gerontology, University of Manchester, Salford Royal Hospital, Salford, UK; Andrology Unit, United Laboratories of Tartu University Clinics, Tartu, Estonia; Department of Andrology and Endocrinology, Katholieke Universiteit Leuven, Leuven, Belgium; NIHR Manchester Musculoskeletal Biomedical Research Unit, Central Manchester NHS Foundation Trust, Manchester Academic Health Sciences Centre, Manchester, UK

**Keywords:** Chronic Widespread pain, Chronic Pain, Vitamin D, Epidemiology, Obesity, Depression

## Abstract

**Background:**

The association between low levels of vitamin D and the occurrence of chronic widespread pain (CWP) remains unclear. The aim of our analysis was to determine the relationship between low vitamin D levels and the risk of developing CWP in a population sample of middle age and elderly men.

**Methods:**

Three thousand three hundred sixty nine men aged 40–79 were recruited from 8 European centres for a longitudinal study of male ageing, the European Male Ageing Study. At baseline participants underwent assessment of lifestyle, health factors, physical characteristics and gave a fasting blood sample. The occurrence of pain was assessed at baseline and follow up (a mean of 4.3 years later) by shading painful sites on a body manikin. The presence of CWP was determined using the ACR criteria for fibromyalgia. Serum 25-hydroxyvitamin D (25-(OH) D) was assessed by radioimmunoassay. Logistic regression was used to determine the relationship between baseline vitamin D levels and the new occurrence of CWP.

**Results:**

Two thousand three hundred thirteen men, mean age 58.8 years (SD = 10.6), had complete pain and vitamin data available and contributed to this analysis. 151 (6.5 %) developed new CWP at follow up and 577 (24.9 %) were pain free at both time points, the comparator group. After adjustment for age and centre, physical performance and number of comorbidities, compared to those in upper quintile of 25-(OH) D ( ≥36.3 ng/mL), those in the lowest quintile (<15.6 ng/mL) were more likely to develop CWP (Odds Ratio [OR] = 1.93; 95 % CI = 1.0-3.6). Further adjustment for BMI (OR = 1.67; 95 % CI = 0.93-3.02) or depression (OR = 1.77; 95 % CI = 0.98-3.21), however rendered the association non-significant.

**Conclusions:**

Low vitamin D is linked with the new occurrence of CWP, although this may be explained by underlying adverse health factors, particularly obesity and depression.

## Background

Musculoskeletal pain (MSP) is a leading cause of morbidity and presentation to primary care [1–4]. In the United Kingdom, 37 % of women and 31 % of men report some kind of chronic pain [5] and approximately 10 % report chronic widespread pain (CWP) [3]. The aetiology of CWP is complex and somewhat poorly understood though both physical and psychological factors have been associated with its development [6–12].

Osteomalacia, a disorder due to profound vitamin D deficiency, is associated with widespread musculoskeletal pain. It remains unclear whether less severe forms of deficiency are also linked with musculoskeletal pain. Vitamin D is obtained either from diet or by synthesis in the skin following UVB light exposure. Prior to becoming biologically active it undergoes two hydroxylations, firstly to 25-hydroxyvitamin D (25-(OH) D) and subsequently to the biologically active form, 1,25 dihydroxyvitamin D (1,25-(OH) _2_D. 25-(OH) D has a long half-life and is used as an indicator of vitamin D status [13].

Results of observational studies assessing the relationship between vitamin D and musculoskeletal pain are somewhat inconsistent, with some, though not all, reporting a significant association between low vitamin D and CWP in specific groups [8, 14, 15]. Similarly in fibromyalgia, a sub-type of CWP, some studies suggest an association with vitamin D deficiency [16, 17] whilst others do not [18, 19]. Using cross-sectional data from the baseline arm of the European Male Ageing Study (EMAS), a multicentre cohort study designed to assess the incidence and prevalence of a range conditions associated with male ageing, we reported an association between CWP and low vitamin D in men at baseline, though the effect was attenuated after adjustment for age, physical activity level, lifestyle factors and depression [15]. There are to our knowledge, however, no data from prospective studies. Experimental studies assessing the effects of vitamin D supplementation on patients’ chronic pain results are also conflicting: a Cochrane review, incorporating trials assessing the effects of vitamin D supplementation on participants with and without vitamin D deficiency, found insufficient evidence of a role for vitamin D supplementation in the treatment of chronic pain [20]. Thus the link between low vitamin D and CWP remains uncertain.

Using data from the prospective arm of EMAS, the aim of this study was to determine i) whether low vitamin D levels were associated with an increased risk of developing CWP in those without CWP, ii) whether low vitamin D was linked with changes in number of reported painful sites and iii) whether any of the apparent associations could be explained by adverse lifestyle or health factors.

## Methods

### Participants & recruitment

Three thousand three hundred sixty nine men were recruited for participation in a population study of male ageing, the European Male Ageing Study (EMAS). Details of the study have been reported previously [21]. Briefly men aged between 40 and 79 years were recruited between 2003–2005 from local population registers across eight European centres, Florence (Italy), Leuven (Belgium), Lodz (Poland), Malmo (Sweden), Manchester (UK), Santiago de Compostela (Spain), Szeged (Hungary) and Tartu (Estonia). Stratified random sampling was used to generate equal numbers of subjects in four 10 year age groups, 40–49, 50–59, 60–69 and 70–79. Participants were invited to take part by letter of invitation which included a postal questionnaire, and an invitation to attend a local clinic for further assessment. The number of subjects recruited varied slightly by centre (range 396–451). Those who attended completed a series of interviewer-assisted questionnaires, had an assessment of physical performance, height and weight and gave a fasted blood sample. 41 % (range 24.1 %-61.5 %) of those invited fully participated in the baseline study by completing both the postal questionnaire and attending the assessment centre. Ethical approval was obtained in each of the centres (Florence-Ethical Committee of the Azienda Ospedaliera Careggi, Leuven-Commissie Medische Ethiek UZ Gasthuisberg, Lodz-Bioethical Committee of Medical University of Lodz for Human Studies, Malmo-Ethical Committee of Lund University, Manchester-North West Multi Centre Ethical Research Committee, Santiago de Compostela-Comité Ético de Investigación Clínica de Galicia, Szeged-Human Investigation Review Board, University of Szeged, Tartu-Ethical Committee, Medical University of Tartu) in accordance with local procedures and all participants gave written informed consent.

### Follow up

Participants were invited to attend for repeat assessment after a mean interval of 4.3 years (range 3–5.7 years). During this time there were 193 deaths. 3176 surviving participants were invited to take part in the follow up stage of the study and 2736 (86 %) did so. Further details of the cohort characteristics are reported elsewhere [22].

### Baseline assessment

The baseline postal questionnaire included questions about lifestyle, including smoking and frequency of alcohol consumption and outdoor exercise [21]. Participants were asked how many days a week they consumed alcohol (response set = never, <1, 2–3, 3–4, 5–6 and > 7 days per week). Smoking status was assessed by were asking whether participants had ever smoked at least 100 cigarettes or been a regular pipe or cigar smoker. Those answering yes to any of the questions were considered as ever smokers. Outdoor exercise was assessed by asking how long participants typically spent outdoors walking or cycling each day (response set = <30 minutes, 30–60 minutes and >60 minutes).

Participants were asked to self-report in the postal questionnaire a range of 16 comorbid conditions which included whether they had ever been diagnosed with cancer, told by a doctor they had a stroke and if they were currently receiving treatment for epilepsy, hypertension, chronic bronchitis, asthma, peptic ulcers, diabetes and heart, liver, kidney, prostate, adrenal, pituitary, thyroid or testicular disease. They were also asked if they had ever fractured a bone after the age of 25. At their clinic visit participants completed an interviewer-assisted questionnaire which included the 21-point Beck Depression Inventory [23] (with scores ≥10 coded as indicating the presence of ‘depression’) and Physical Activity in the Elderly (PASE) score [24]. Participants completed a 50 foot walking test and also a sit to stand test with the time taken to complete each task recorded in seconds [25, 26]. Height and weight were also measured and body mass index (BMI) was calculated as weight in kilograms divided by height in metres squared.

### Assessment of pain

**Fig. 1:**
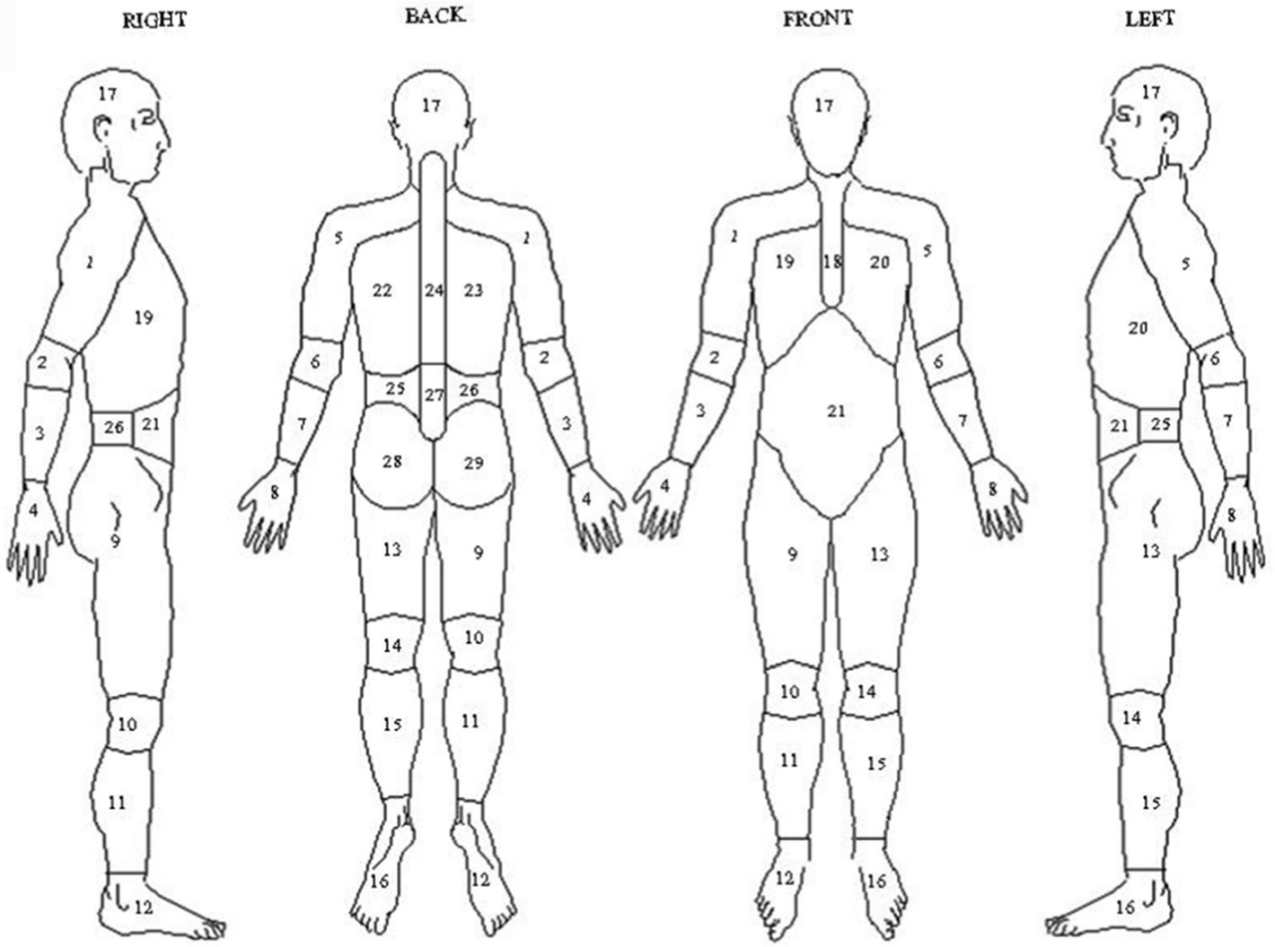
Four view body mannequin, with imposed 29 region coding frame, on which participants were asked to shade the site of their pain

At baseline and follow up in the postal questionnaire participants were asked ‘In the past month have you had any pain which has lasted for one day or more? Those who reported experiencing pain were asked to shade where they experienced pain on a 4 view body mannequin, Fig. 1. To assess chronicity they were asked ‘Thinking about this ache or pain, have you been aware of it for more than 3 months?’ Pain was then coded to number of pain sites using a 29-region coding frame [27]. Three groups were defined: those experiencing no pain, those with some pain and those with CWP. The latter defined as those meeting the American College of Rheumatology 1990 criteria for fibromyalgia [28]: pain involving the axial skeleton, both sides of the body and above and below the waist lasting for at least 3 months. This method to assess pain has been used widely in population studies and has construct validity [27, 29, 30]. Participants who met the criteria for CWP at follow up but not baseline were coded as having developed new CWP.

### Biochemical assays

At baseline, following an overnight fast, a blood sample was taken and the serum was frozen at−80 °C and sent on dry ice to a single laboratory for analysis (University of Leuven). Serum levels of 25-(OH) D, as the recommended marker of vitamin D status [31], were determined by radioimmunoassay (DiaSorin; Stillwater, Minnesota, USA) with results expressed as ng/mL. Intra-and inter-assay coefficients of variation (CV) for 25-(OH) D were 11 % and 8 %, respectively. The detection limit of the radioimmunoassay kit for 25-(OH) D was 1.5 ng/ml. We also assessed the serum levels of 1,25-dihydroxyvitamin D (1,25-(OH) _2_D) as this is the biologically active form of vitamin D. Serum 1, 25-(OH) _2_D levels were measured using high performance liquid chromatography-tandem mass spectrometry as described by Casetta et al. [32] and modified by Vanderschueren et al. [33] with results expressed as pg/mL. Ultra pure methanol (Optima liquid chromatography/mass spectrometry, Fisher Scientific) was used for protein precipitation of 200 μL serum samples. 180 μl of supernatant was injected on a Shimadzu Prominence HPLC (Shimadzu, Kyoto, Japan) coupled to a AB Sciex API 5500 QTRAP tandem mass spectrometer (Sciex, Warrington, UK). The limit of quantification was <6.25 pg/mL. The inter-day imprecision of pooled serum at high and low serum 1,25-(OH) _2_D concentrations was assessed. For serum with a low concentration (mean 7.16 pg/mL) the CV was 10.1 % and 5.9 % for serum with a high concentration (mean 55.8 pg/mL).

### Statistical analysis

Descriptive statistics were used to describe the characteristics of the study sample. Depending on their distribution parametric (t-tests) and nonparametric (wilcoxon rank sum) tests to determine the significance of any differences in any of the continuous variables between those who developed new CWP at follow up and those who were pain free at both time points. χ^2^ tests were used to test differences in categorical data. 25-(OH) D and 1,25-(OH) _2_D levels were categorised into quintiles. We used logistic regression to determine the association between the new occurrence of CWP and 25-(OH) D and 1,25-(OH) _2_D with those in the highest quintile as the reference category. Adjustments were made for factors which significantly differed between those with new CWP at follow up and those who remained pain free throughout. This included age and centre and subsequently physical performance (time to sit from standing and time to walk 50 feet), number of comorbidities, BMI and the presence of depression (determined by BDI ≥10). We also assessed the relationship between vitamin D deficiency and CWP with 25-(OH) <20 ng/mL considered to represent deficiency [13]. We looked also at the number of painful sites (out of 29) at baseline and follow up. Because of the large number of participants with no painful sites (*N =* 962) zero-inflated negative binomial regression was performed to assess the relationship between vitamin D and the numbers of pain sites reported at follow up. Adjustments were made for age and centre and subsequently for physical performance (time to sit from standing and time to walk 50 feet), number of comorbidities, BMI and the presence of depression (determined by BDI ≥10). To limit the analysis to change in number of pain sites over the follow up period we also adjusted for the number of pain sites reported at baseline. Results of the logistic regression were presented as Odds Ratios (OR) and for zero-inflated negative binomial regression as Incidence Rate Ratio (IRR); both with 95 % confidence intervals. Statistical analysis was performed using Stata SE 11.2 (Stata Corp, College Station, Texas, USA).

## Results

### Participants

Two thousand seven hundred thirty six men participated in both the baseline and follow up phases of EMAS. Of these 2313 (84.5 % of those who participated in both phases and 68.7 % of those in the original cohort) had complete data for 25-(OH) D and pain at baseline and follow up and were included in this analysis. Measurement of vitamin D was undertaken during the; summer *N =* 269 (11.6 %), autumn *N =* 816 (35.3), winter *N =* 701 (30.3 %) and spring *N =* 527 (22.8 %). The mean age of these participants at baseline was 58.8 years (standard deviation [SD] = 10.6 years).

### Baseline characteristics

Baseline characteristics of the 2313 with complete data are presented in Table 1. Mean BMI was 27.6 Kg/m^2^ [SD = 4.0]. 57.4 % of men consumed alcohol at least once a week, 69.7 % had ever smoked and 11.8 % were depressed. 60.9 % had at least one comorbid condition and 41.1 % two comorbid conditions. Mean 25 (OH) D, and 1,25 (OH) _2_D levels, were 25.9 ng/mL [SD = 12.6] and 59.4 pg/ml [SD = 15.9], respectively. 195 (8.4 %) participants fulfilled the criteria for CWP, 1159 (50.1 %) had some pain (pain which did not meet the CWP criteria) and 959 (41.5 %) were pain free. The median number of pain sites was 1 (IQR 0–4).

**Table 1:** Subject characteristics at baseline for all subjects (*N =* 2,313)

Subject characteristics	
	mean (SD)
Age (years)	58.8 (10.6)
Body mass index (kg/m^2^)	27.6 (4.0)
Sit to stand time (seconds)	12.4 (3.2)
Time to walk 50 feet (seconds)	13.2 (2.8)
PASE score	201.2 (88.4)
25 (OH) D (ng/mL)	25.9 (12.6)
1,25 (OH) _2_ D (pg/mL)	59.4 (15.9)
Alcohol-number of days alcohol consumed	*N* (%)
Never	349 (15.1)
<1 week	631 (27.4)
1–2	489 (21.2)
3–4	297 (12.8)
5–6	171 (7.4)
7	371 (16.1)
Ever smoked (vs. never smoker)	1612 (70.8)
Depression^a^ (vs. not depressed)	272 (11.9)
Number of comorbidities	
None	893 (39.1)
1	453 (19.8)
≥2	940 (41.1)
Self-reported ≥30 minutes walking/cycling outside per day (vs. <30 minutes)	1493 (64.8)
Pain status	
Pain free	959 (41.5)
Some pain	1159 (50.1)
CWP	195 (8.4)
	median (IQR)
Number of pain sites	1 (0–4)

PASE, Physical Activity Scale for the Elderly; 25 (OH) D, 25-hydroxyvitamin D3; 1,25 (OH) 2 D, 1,25-dihydroxyvitamin D3. ^a^The presence of depression was determined using Beck Depression Inventory with scores ≥10 indicating the presence of depression

### Pain status at follow up

Overall 223 (9.6 %) participants fulfilled the criteria for CWP at follow up, 1140 (49.3 %) had some pain and 950 (41.1 %) reported no pain. Of the 2118 men without CWP at baseline 151 (7.1 %) developed new onset CWP at follow up. 577 participants (25.0 %) were pain free at both baseline and follow up. Overall at follow up pain status was different from baseline for 980 (42.4 %) participants, see Table 2.

**Table 2:** Change in pain status between baseline and follow up

		Pain status at follow up			Total
		Pain free	Some pain	Chronic widespread pain	
Pain status at baseline	Pain free	577	355	27	959
					(41.5)
	Some pain				
		351	684	124	1159
					(50.1)
	Chronic widespread pain				
		22	101	72	195
					(8.4)
	Total	950	1140	223	2313
		(41.1)	(49.3)	(9.6)	

All values are *N* (%)

### Factors linked with the new occurrence of CWP

Compared to those without pain at baseline or follow up those who developed new onset CWP (*N =* 151) were more likely to have a higher BMI (28.6 vs. 27.2 Kg/m^2^; *p <* 0.001) and were more likely to have a BDI score of ≥10 indicating the presence of depression (23.5 % vs. 5.6 %; *p <* 0.001), see Table 3. They were also more likely to have at least 2 self-reported comorbidities, have a longer sit to stand time (13.3 vs. 12.2 seconds; *p <* 0.001) and time to walk 50 feet (14.2 vs. 12.9 seconds; *p <* 0.001). There was no significance difference observed in their 25 (OH) D (24.2 vs. 26.3 ng/mL; P = 0.07) or 1,25 (OH) D (58.2 pg/mL vs. 60.2 pg/mL; P = 0.21) levels. There was also no significant difference in their PASE score, level of self-reported outdoors activity, smoking or alcohol consumption.

**Table 3:** Subject characteristics at baseline by pain status

Subject characteristics	Pain free at baseline and follow up (*N =* 577)	New onset chronic widespread pain at follow up (*N =* 151)
	mean (SD)	mean (SD)
Age (years)	58.9 (10.7)	59.3 (10.5)
Body mass index (kg/m^2)^	27.2 (3.8)	28.6 (4.2)‡
Sit to stand time (seconds)	12.2 (3.0)	13.3 (4.0)‡
Time to walk 50 feet (seconds)	12.9 (2.2)	14.2 (4.7)‡
PASE score	196.0 (94.4)	194.8 (89.1)
25 (OH) D (ng/mL)	26.3 (12.2)	24.2 (12.2)
1,25 (OH) _2_D (pg/mL)	60.2 (16.3)	58.2 (17.2)
Alcohol-number of days alcohol consumed	***N (%)***	***N (%)***
Never	82 (14.2)	34 (22.7)
<1 week	166 (28.8)	38 (25.3)
1–2	124 (21.5)	29 (19.3)
3–4	66 (11.4)	21 (14.0)
5–6	38 (6.6)	11 (7.3)
7	101 (17.5)	17 (11.3)
Ever smoked (vs. never smoker)	377 (66.6)	110 (73.3)
Depression^a^ (vs. not depressed)	32 (5.6)	35 (23.5)†
Number of comorbidities		
None	272 (47.1)	37 (24.5)
1	108 (18.7)	25 (16.6)
≥2	197 (34.1)	89 (58.9)†
Self-reported ≥30 minutes walking/cycling outside per day (vs. <30 minutes)	382 (66.4)	99 (65.6)

PASE, Physical Activity Scale for the Elderly; 25 (OH) D, 25-hydroxyvitamin D3; 1,25 (OH) 2 D, 1,25 dihydroxyvitamin D3. *The presence of depression was determined using Beck Depression Inventory with scores ≥10 indicating the presence of depression. †Chi squared test *p <* 0.05, ‡T test *p <* 0.05

### Vitamin D and CWP

After adjustment for age and centre, compared to those in the upper quintile of 25-(OH) D (>36.3 ng/mL) those in the lowest quintile (<15.6 ng/mL) were more likely to develop CWP (OR = 2.32; 95 % CI = 1.27–4.23), see Table 4. This relationship remained significant after adjustment for physical performance and comorbidity though not after further adjustment for BMI and depression (determined by BDI ≥10), OR = 1.60, 95 % CI = 0.83-3.09. Adjustment for either BMI (OR = 1.67; 95 % CI = 0.93-3.02) or depression (OR = 1.77; 95 % CI = 0.98–3.21), in addition to age and centre, also rendered the relationship not statistically significant. After adjustment for age and centre, compared to those in the upper quintile of 1, 25 (OH) _2_D, those in lower quintiles were more likely to develop CWP though none were statistically significant. We identified no association between CWP and the presence of vitamin D deficiency (25-(OH) D <20 ng/mL) in either the adjusted or unadjusted analysis when compared to those with higher levels (adjusted for age and centre, OR 1.32; 95 % CI 0.91–1.93).

**Table 4:** The association between baseline 25 (OH) D and 1,25 (OH) _2_D and the new occurrence of chronic widespread pain

	**Model 1**	**Model 2**	**Model 3**	**Model 4**
25 (OH) D–quintiles				
1: ≥36.3 ng/mL	Referent	Referent	Referent	Referent
2: 26.7–36.2 ng/mL	1.41 (0.76–2.59)	1.24 (0.66–2.32)	1.23 (0.64–2.33)	1.07 (0.55–2.08)
3: 20.7–26.6 ng/mL	1.43 (0.77–2.65)	1.29 (0.68–2.43)	1.30 (0.68–2.48)	1.18 (0.60–2.30)
4: 15.6–20.6 ng/mL	1.29 (0.68–2.42)	1.17 (0.61–2.23)	1.19 (0.61–2.30)	1.18 (0.60–2.31
5: <15.6 ng/mL	2.32 (1.27–4.23)*	2.03 (1.10–3.75)*	1.93 (1.03–3.62)*	1.60 (0.83–3.09)
1,25 (OH) _2_ D–quintiles				
1: ≥72.5 pg/ml	Referent	Referent	Referent	Referent
2: 62.2–72.5 pg/mL	1.15 (0.60–2.21)	1.05 (0.54–2.04)	0.99 (0.50–1.96)	0.94 (0.46–1.90)
3: 55.2–62.0 pg/mL	1.23 (0.65–2.34)	1.12 (0.58–2.17)	1.12 (0.57–2.20)	1.07 (0.52–2.17)
4: 45.4–55.0 pg/mL	1.37 (0.70–2.65)	1.41 (0.72–2.77)	1.42 (0.71–2.83)	1.33 (0.65–2.73)
5: <45.4 pg/mL	1.79 (0.94–3.42)	1.70 (0.87–3.31)	1.55 (0.78–3.08)	1.52 (0.74–3.11)

Results determined using logistic regression with those in the highest quintile serving as the referent group. Results are expressed as odds ratio (95 % confidence intervals) * *p <* 0.05. 25 (OH) D, 25-hydroxyvitamin D3; 1,25 (OH) _2_D, 1,25 dihydroxyvitamin D3. Model 1, adjusted for age and centre; Model 2, adjusted for age, centre and physical performance (sit to stand time and time to walk 50 feet); Model 3, adjusted for age, centre, physical performance and comorbidities; Model 4, adjusted for age, centre, physical performance, comorbidities, body mass index and depression (the presence of depression was determined using Beck Depression Inventory with scores ≥10 indicating depression)

### Vitamin D and number of pain sites

At baseline those with CWP at follow up had median 8 (IQR 6–11) painful sites and those with some pain had 3 (IQR 2–5). After adjusting for age and centre, compared to those in the upper quintile of vitamin D, those in the 5th and 2nd quintile were more likely to report more pain sites (5th quintile IRR = 1.2; 95 % CI = 1.0–1.4; 2nd quintile IRR 1.2 95 % CI 1.0–1.3), see Table 5. After adjustment for number of pain sites at baseline these associations became non-significant. Further adjustment for physical performance, comorbidity, BMI and depression (presence determined by BDI ≥10) attenuated slightly the IRRs. There was no significant relationship observed between 1,25 (OH) _2_D and number of pain sites.

**Table 5:** The association between baseline 25 (OH) D and 1,25 (OH) _2_D and the number of painful sites at follow up

	**Model 1**	**Model 2**	**Model 3**	**Model 4**
25 (OH) D-quintiles				
1: ≥36.3 ng/mL	Referent	Referent	Referent	Referent
2: 26.7–36.2 ng/mL	1.16 (1.00–1.34)*	1.12 (0.98–1.28)	1.11 (0.97–1.26)	1.13 (0.99–1.29)
3: 20.7–26.6 ng/mL	1.05 (0.91–1.22)	1.05 (0.92–1.21)	1.03 (0.90–1.19)	1.03 (0.90–1.18)
4: 15.6–20.6 ng/mL	1.12 (0.96–1.30)	1.12 (0.98–1.29)	1.12 (0.97–1.28)	1.12 (0.97–1.28)
5: <15.6 ng/mL	1.20 (1.03–1.40)*	1.14 (0.99–1.31)	1.11 (0.97–1.28)	1.10 (0.95–1.26)
1,25 (OH) _2_ D-quintiles				
1: ≥72.5 pg/ml	Referent	Referent	Referent	Referent
2: 62.2–72.5 pg/mL	0.95 (0.81–1.11)	0.93 (0.81–1.07)	0.92 (0.80–1.06)	0.93 (0.81–1.07)
3: 55.2–62.0 pg/mL	0.98 (0.84–1.14)	0.99 (0.86–1.14)	0.98 (0.85–1.13)	1.00 (0.86–1.15)
4: 45.4–55.0 pg/mL	1.06 (0.91–1.23)	1.09 (0.95–1.26)	1.10 (0.96–1.27)	1.10 (0.95–1.26)
5: <45.4 pg/mL	1.03 (0.88–1.21)	1.10 (0.88–1.17)	1.04 (0.90–1.20)	1.03 (0.89–1.19)

Results determined using zero-inflated negative binomial regression with those in the highest quintile serving as the referent group. Results are expressed as Incidence rate ratio (95 % confidence intervals) * *p <* 0.05. 25-(OH) D, 25-hydroxyvitamin D3; 1,25-(OH) _2_D, 1,25 dihydroxyvitamin D3. Model 1, adjusted for age and centre; Model 2, adjusted for age, centre and number of pain sites at baseline; Model 3, adjusted for age, centre, and number of pain sites at baseline and physical performance (sit to stand time and time to walk 50 feet); Model 4, adjusted for age, centre, and number of pain sites at baseline, physical performance, number of comorbidities, body mass index and depression (the presence of depression was determined using Beck Depression Inventory with scores ≥10 indicating depression)

## Discussion

In this analysis, compared to those in the upper quintile of serum 25 (OH) D level men in the lowest quintile (<15.6 ng/mL) at baseline were more likely to have developed CWP at follow up though this appeared to be related to the presence of adverse health factors, particularly obesity and depression. No significant association was observed between 1,25 (OH) _2_D and the new occurrence of CWP.

Some previous observational and experimental studies have suggested that low levels of vitamin D may have a causal role in the development of chronic pain, though the evidence is conflicting [20]. We however observed, after adjustment, no independent association between vitamin D at baseline and CWP at follow up in our male, and largely Caucasian, population and our results are in keeping with previous studies which have found no association either at all or not in men [8, 15, 18], though a significant association has been reported previously in women [8]. There is also some evidence of ethnic variation in the prevalence of CWP with higher rates observed in non-caucasians [14, 34] and in some ethnic groups, such as British residents of South Asian ethnic origin the prevalence of both vitamin D deficiency and CWP are significantly increased [14]. It is therefore possible that whilst we did not observe an independent association between low vitamin D and the development of CWP in our population the pathogenesis of CWP may be different in specific groups, and in some this may include low vitamin D.

We also assessed the association 1,25-(OH) _2_D, the biologically active form of vitamin D and CWP. We observed a trend towards an increased risk of CWP for those in the lowest quintile of 1,25-(OH) _2_D, though in contrast to our findings with 25-(OH) D, even in the unadjusted analysis this did not reach statistical significance. 1,25-(OH) _2_D has a short half-life and the serum level is dependent on a range of other factors including serum phosphate, calcium and parathyroid hormone levels. Current guidance recommends 1,25-(OH) _2_D is not useful in determining the vitamin D status of patients [31] and therefore this measure likely poorly reflects the vitamin D status of our population.

Approximately half of the study participants at either baseline or follow up reported experiencing some pain, though did not meet the criteria for CWP. This is a heterogeneous group ranging from those with localised pain to those almost meeting the criteria for CWP. It seems unlikely, however, that inclusion or reclassification of those with more severe pain (within the ‘some pain’ group) would have influenced the findings.

In our population 8.4 % and 9.6 % of participants met the criteria for CWP at baseline and follow up respectively, which is higher than the prevalence of CWP previously reported in community-dwelling men [3, 4]. We observed significant changes in pain status between baseline and follow up with some 42.4 % of participants changing pain status which is broadly consistent with findings from other studies [35, 36].

We observed a strong association between depression, the presence of which was determined using the BDI, and CWP. This has been observed in other studies and is consistent with reports of the NHANES-1 cohort [37] in the USA which found depression predicted the risk of developing chronic musculoskeletal pain. We also observed that increased BMI was a predictor of CWP in keeping with the co-occurrence of CWP and raised BMI reported previously [38], although this is the first prospective study to demonstrate a link. Smoking and alcohol consumption were not however linked with CWP in contrast to previous reports [39].

There are a number of limitations which need to be considered in interpreting our data. The participation rate for the baseline survey was 41 % of those invited to take part. There were significant differences between those who participated in the full study (*N =* 3369) and those who returned the initial postal questionnaire only (*N =* 594), most relevant of which was the prevalence of pain. 59.4 % of full study participants reported pain lasting one day or more in the preceding month at baseline compared to 43.8 % of those who returned the questionnaire only [21]. Such potential selection factors mean that caution is needed in interpreting the absolute prevalence of pain observed, however, they are unlikely to have influenced our main findings of a link between CWP and vitamin D which were based on an internal comparison of those recruited. In our study pain status was assessed prospectively after an interval of 4.3 years following assessment of vitamin D. Any change in vitamin D status during that time would result in misclassification of D status and reduce the likelihood of findings significant associations. In a study though of postmenopausal women there was only moderate intra-individual variation in serum 25-(OH) D between baseline and follow up at 5 years and the authors supported the use of single point measurement of vitamin D in prospective studies with follow up periods less than 5 years [40]. Vitamin D is synthesised in the skin, dependant on skin type and levels of UVB exposure, however, we did not have any information about these factors in our data, and were not therefore able to make adjustment for them. There is also variation in levels of D during the year with higher levels during the summer months–we observed however no association between CWP and season (data not shown) and so did not make any adjustment for season. It is also possible that any effect of vitamin D on the development of CWP occurs through a mediator variable, such as depression [41], which we treated as a confounder and adjusted for in our analysis thereby potentially masking a true association. Additionally, physical activity and performance was assessed using the PASE score, time to stand from sitting and also time to walk 50 feet. Though widely used, these tests have not been validated across the entire age range included in this study. Finally the men who contributed data to this analysis were predominantly European and Caucasian and the results should be extrapolated beyond this with caution.

## Conclusions

In this population study men with very low vitamin D (25-(OH) D <15.6 ng/mL) at baseline were at significantly increased risk of having developed CWP at follow up, though this appears to be related to other adverse health factors, particularly raised BMI and depression.

## Abbreviations

1,25 (OH)_2_D: 1,25-dihydroxyvitamin D
25 (OH) D: 25-hydroxyvitamin D
BMI: body mass index
CWP: chronic widespread pain
EMAS: European male ageing study
IRR: incidence rate ratio
OR: odds ratio
